# The Isolated Mouse Jejunal Afferent Nerve Assay as a Tool to Assess the Effect of Botulinum Neurotoxins in Visceral Nociception

**DOI:** 10.3390/toxins14030205

**Published:** 2022-03-11

**Authors:** Kevin Retailleau, Vincent Martin, Stephane Lezmi, Camille Nicoleau, Jacquie Maignel

**Affiliations:** IPSEN Innovation, 5 Avenue du Canada, 91940 Les Ulis, France; vincent.martin@ipsen.com (V.M.); stephane.lezmi@ipsen.com (S.L.); camille.nicoleau@ipsen.com (C.N.); jacquie.maignel@ipsen.com (J.M.)

**Keywords:** visceral pain, botulinum neurotoxin A, afferent nerve assay, distension, capsaicin, ex vivo model

## Abstract

For the past two decades, botulinum neurotoxin A (BoNT/A) has been described as a strong candidate in the treatment of pain. With the production of modified toxins and the potential new applications at the visceral level, there is a real need for tools allowing the assessment of these compounds. In this study, we evaluated the jejunal mesenteric afferent nerve assay to investigate BoNT/A effects on visceral nociception. This ex vivo model allowed the continuous recording of neuronal activity in response to various stimuli. BoNT/A was applied intraluminally during three successive distensions, and the jejunum was distended every 15 min for 3 h. Finally, samples were exposed to external capsaicin. BoNT/A intoxication was validated at the molecular level with the presence of cleaved synaptosomal-associated protein of 25 (SNAP25) in nerve terminals in the mucosa and musculosa layers 3 h after treatment. BoNT/A had a progressive inhibitory effect on multiunit discharge frequency induced by jejunal distension, with a significant decrease from 1 h after application without change in jejunal compliance. The capsaicin-induced discharge was also affected by the toxin. This assay allowed the description of an inhibitory effect of BoNT/A on afferent nerve activity in response to distension and capsaicin, suggesting BoNT/A could alleviate visceral nociception.

## 1. Introduction

Famous in the field of aesthetics, botulinum neurotoxin A (BoNT/A), produced by the bacterium *Clostridium botulinum*, is also a therapeutic solution in the treatment of various pathologies [1]. The main effect of this neurotoxin is on peripheral cholinergic synapses, inducing the inhibition of acetylcholine release. In brief, after binding onto a synaptic vesicle 2 (SV2) receptor and undergoing cellular internalization, BoNT/A cleaves SNAP25 (Synaptosomal-Associated Protein, 25 kDa) [2]. This membranal t-SNARE protein (Target Soluble NSF (*N*-ethylmaleimide-sensitive factor) Attachment Protein Receptor), as is characteristic of the other SNARE family members, plays an important role in the membrane fusion mechanism [3]. Thus, cleavage of this protein causes the inhibition of neurotransmitter exocytosis [4]. Since its first regulatory approval by the US FDA in 1989 for the treatment of strabismus and blepharospasm [5], BoNT/A, due to its inhibitory effect on acetylcholine release at the neuromuscular junction level, has become a solution for the management of an increasing number of diseases, such as movement disorders (hemifacial spasm and other spastic disorders, focal dystonia, etc.) [6,7,8,9]. Subsequently, the use of BoNT/A was extended to the treatment of non-muscular diseases. For example, injections of BoNT/A are also used to treat hyperhidrosis and sialorrhea, by preventing the hyperstimulation of eccrine sweat and salivary glands, respectively [1,10,11,12].

Since the 2000s, this neurotoxin has appeared as a strong candidate drug in the treatment of pain, with the publication of several studies describing BoNT/A as having an effect in different pain models [13]. Even though the mechanism of action of BoNT/A at the afferent/sensory fiber level is still under investigation, its analgesic effects are now recognized, permitting its use in treating various painful conditions, such as diabetic neuropathy, joint pain, and migraine [14].

With the increasing number of potential new applications in pain relief, especially at the visceral level (e.g., for interstitial cystitis, endometriosis) [15,16], and the development of new technologies for the production of modified toxins [17], there is a need for tools allowing the assessment of these new compounds. However, pain perception is a complex multifactorial process involving afferent nerves, spinal neurons, ascending and descending pathways of facilitation and inhibition, and several different brain regions [18,19,20]. It is now also known that the psychological aspect is very important in the way pain is perceived [21]. Changes in any of these elements can affect sensory signals and result in the generation or modification of pain [18]. All these aspects make it difficult to assess the effect of a compound on pain in vivo [22]. In the case of visceral pain, this is complicated by specific phenomena, such as cross-organ sensitization and pain-referred mechanisms [23,24], which further complicate the identification of sources of visceral pain and, therefore, the evaluation of the impact of such compounds as botulinum neurotoxin.

An ex vivo approach is an effective way to assess the effect of such compounds in an organ-specific manner without the influence of other nervous structures implicated in pain integration (dorsal root ganglion, spinal cord, and brain). In order to understand the molecular mechanisms involved in visceral sensory perception, or to evaluate the effect of compounds on visceral nociception, ex vivo models of visceral afferent recording have been developed. For example, an assay allowing the measurement of afferent nerve activity in an isolated bladder was used to establish the properties of mechanosensory neurons projecting to the bladder, and to understand the neural mechanisms of lower urinary tract symptoms in obstruction-induced bladder overactivity [25,26]. This assay was also used to describe the inhibitory effect of onabotulinum toxin A on bladder afferent nerve activity [27]. Similarly, the jejunum mesenteric nerve assay, described in detail by Nullens et al. [28], has also been used to understand the mechanisms involved in jejunal signal transduction in response to mechanical and chemical stimuli. This ex vivo model provides the opportunity to continuously measure the mesenteric nerve activity in response to stimuli without modulation or input from the central nervous system. Thus, the transient receptor potential vanilloid 1 channel (TRPV1) has been described as a component effecting jejunal afferent sensitivity to distension and acidity in mice [29]. It is therefore interesting to evaluate the impact of BoNT/A on the afferent nerve activity of another visceral organ, such as the intestine. In addition, a significant advantage of working with the jejunum is the possibility of using several jejunal segments from the same animal [28]. This makes it possible to compare the effects of different toxins on the same animal; furthermore, from an ethical point of view, it reduces the number of animals used.

The main objectives of this study were to evaluate whether the jejunum mesenteric nerve assay could be an effective tool to assess the effects of toxins on visceral nociception, and to measure the impact of an intraluminal perfusion of a controlled quantity of BoNT/A on mesenteric afferent nerve activity in response to nociceptive jejunal distension and capsaicin serosal exposure.

## 2. Results

### 2.1. The Jejunum Mesenteric Nerve Assay: A Sensitive Assay

**Figure 1:**
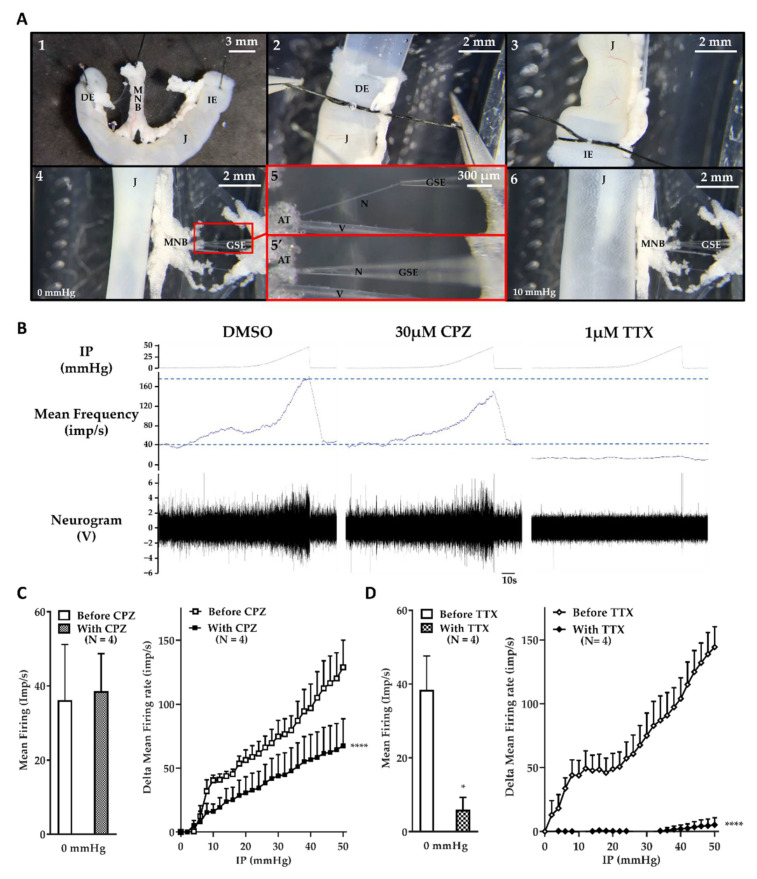
Effect of reference compounds on afferent firing frequency. (**A**) Illustration of jejunum/nerve segment mounting. (1) The jejunum (J) was cut in approximately 3 cm long loops with a mesenteric neurovascular bundle (MNB), and the duodenal (DE) and the ileal ends (IE) were identified. Each segment was flushed with KH solution to remove luminal contents. (2) The duodenal end (DE) was mounted on the input tube and firmly fixed with a knot. (3) The other end of the jejunum (IE) was mounted on the output tube and secured with a knot. (4) The jejunum was slightly stretched in order to limit jejunal deformation during the distension, taking care not to exert excess tension. The MNB was pinned to facilitate nerve dissection. (5) Magnification of the red box, the nerve (N) was isolated from the vein (V) and artery and aspirated with glass suction electrode (GSE). (5′) Some adipose tissue (AT) was aspirated into the glass capillary to induce mechanical ‘sealing’. (6) Example of a jejunal distension induced by an IP of 10 mmHg. (**B**) Example of multiunit discharge recorded in response to jejunal distension on the same sample under control condition (dimethyl sulfoxide, DMSO; left side), after 5-min incubation with 30 µM capsazepine (CPZ; middle), and after 5-min incubation with 1 µM tetrodotoxin (TTX; right side). The internal pressure (IP; in millimeters of mercury, mmHg) is presented in the top panel. The firing frequency (impulses per second, imp/s) is presented in the middle panel, and in the bottom panel is the multiunit recording (i.e., a neurogram) (in volts, V). (**C**) Quantification of the afferent firing frequency measured under basal conditions (left graph; IP = 0 mmHg) and in response to distension (right graph; IP = 0 to 50 mmHg) before and after 5-min incubation with 30 µM CPZ. (**D**) Quantification of the afferent firing frequency measured under basal conditions (left graph; IP = 0 mmHg) and in response to distension (right graph; IP = 0 to 50 mmHg) before and after 5-min incubation with 1 µM TTX. The results are presented as mean firing rate (imp/s) under basal conditions and in delta mean firing rate (imp/s) as a function of internal pressure, and correspond to the average of mean or delta mean firing frequency obtained under basal conditions or at each IP, respectively. Number (N) of mice is indicated by 1 jejunum segment per mouse. Data are presented as mean ± standard error of the mean (SEM) from 4 independent experiments for each treatment (* *p* < 0.05; **** *p* < 0.0001; paired Student’s *t*-test; compound vs. buffer).

The sensitivity of the jejunum mesenteric nerve assay was evaluated with the use of two inhibitors targeting channels known to be involved in mechanosensitive multiunit discharge (Figure 1). The first was capsazepine (CPZ), a specific TRPV1 channel antagonist [30]. TRPV1, a non-selective cation channel, was described as a component of mechanical visceral nociception [29,31,32]. Inhibition of this channel with CPZ induces a partial inhibition of the multiunit discharge frequency in response to jejunal distension [29]. The second was tetrodotoxin (TTX), a specific and potent blocker of voltage-dependent sodium channels [33,34,35]. This toxin was observed to block visceral afferent mechanosensitivity [36]. As illustrated (Figure 1A), each jejunal segment was mounted between two tubes and subjected to successive distensions with or without an inhibitor. The aspiration of the whole nerve into a glass suction electrode allowed the continuous recording of multiunit discharge, i.e., action potentials generated by all fibers composing the nerve.

After 5-min incubation with 30 µM CPZ in the external medium, the basal multiunit discharge frequency, measured at an intraluminal pressure (IP) of 0 mmHg, was not affected by the antagonist, with a spontaneous firing frequency of 36.15 ± 7.52 imp/s and 38.6 ± 10.2 imp/s with dimethyl sulfoxide (DMSO) and CPZ, respectively (*p* = 0.59; Figure 1B,C). However, the multiunit discharge frequency measured in response to jejunal distension with CPZ was significantly (*p* < 0.0001) lower than previous responses in the presence of DMSO. The delta mean firing frequency in response to a low IP, such as 10 mmHg, was 40.5 ± 4.1 imp/s under control conditions (DMSO) and 16.2 ± 5.7 imp/s in the presence of CPZ, and was 128.8 ± 21.3 vs. 67.5 ± 21.1 imp/s at a high IP, such as 50 mmHg, with DMSO and CPZ, respectively (Figure 1C). The inhibition of TRPV1 induced a partial inhibition of the multiunit discharge induced by a mechanical stimulation.

In addition, the 5-min incubation with 1 µM TTX in the external medium caused a significant (*p* = 0.026) decrease in firing frequency under basal conditions (IP = 0 mmHg), with a spontaneous firing frequency of 31.00 ± 8.20 imp/s and 4.79 ± 2.84 imp/s without and with TTX, respectively (Figure 1B,D). Moreover, the multiunit discharge in response to jejunal distension was almost totally and significantly (*p* < 0.0001) inhibited by the blockage of voltage-dependent sodium channels. The average delta mean firing frequency induced by low IP, such as 10 mmHg, was 43.9 ± 11.7 vs. −0.4 ± 0.5 imp/s, and was 144.4 ± 16 vs. 5.2 ± 5.5 imp/s at high IP, such as 50 mmHg, without and with TTX, respectively (Figure 1D). The inhibition of voltage-gated sodium channels induced a strong inhibition of non- and mechanosensitive afferent nerve activity.

In summary, the sensitivity of the jejunum mesenteric nerve assay made it possible to describe both the partial inhibition of the mechanosensitive afferent nerve activity by CPZ and the global inhibition of the afferent nerve activity induced by TTX.

### 2.2. An Effective BoNT/A Intoxication

**Figure 2:**
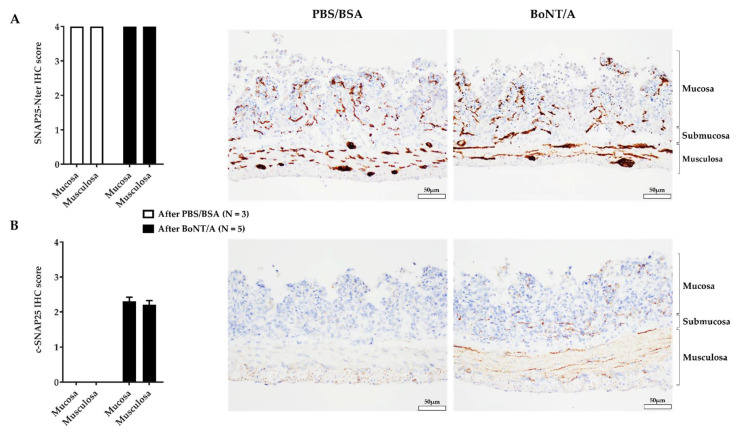
Immunostaining of total and cleaved SNAP25 in jejunum treated with phosphate-buffered saline/ bovine serum albumin (PBS/BSA) or botulinum neurotoxin A (BoNT/A). (**A**) Staining levels (bar graph) and immunochemistry staining of total synaptosomal-associated protein 25 (SNAP25 N-ter) in jejunum 3 h after PBS/BSA (middle) or BoNT/A (right) treatment. (**B**) Staining levels (bar graph) and immunochemistry staining of cleaved SNAP25 (c-SNAP25) in jejunum, 3 h after PBS/BSA (left) or BoNT/A (right) application. Scale bar is 50 µm. Number (N) of mice is indicated by 1 jejunum segment per mouse. Data are expressed as mean ± SEM from 3 or 5 jejunum preparations treated with PBS/BSA or BoNT/A, respectively.

Prior to functional evaluation, the ability of BoNT/A to cleave SNAP25 was assessed to verify the effectiveness of our treatment. To do so, immunostaining of the total and cleaved SNAP25 (c-SNAP25) was performed on sections from BoNT/A- and PBS/BSA (vehicle)-treated jejunum preparations. The levels of SNAP25 N-ter (full and cleaved forms) were similar in PBS/BSA- and BoNT/A-treated samples (Figure 2A). There was staining observed at the nerve endings in the mucosa (villi) and in the musculosa as well as in/around the neuron cell bodies of the submucosal and myenteric plexi. Regarding the cleaved form levels, there was a clear difference. As expected, after PBS/BSA perfusion, c-SNAP25 was not observed in jejunal tissue sections. In contrast, staining of c-SNAP25 occurred in BoNT/A-treated samples. Three hours after intraluminal perfusion of the toxin, c-SNAP25 was only observed in the nerve terminals of the lamina propria and the muscularis; minimal perineuronal staining was also noted in the plexi (Figure 2B).

To conclude, the intraluminal application of BoNT/A during three successive distensions allowed the jejunum/nerve preparation intoxication.

### 2.3. BoNT/A Inhibits Jejunal Multiunit Afferent Nerve Discharge

**Figure 3:**
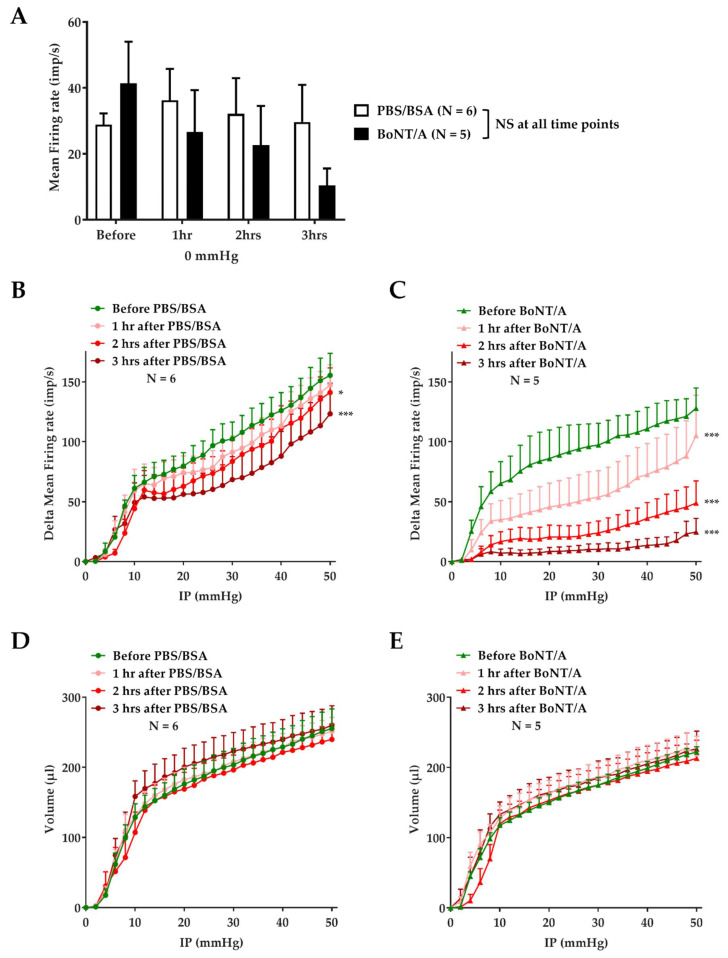
Effect of BoNT/A on afferent firing frequency induced by distension and compliance. (**A**) Quantification of the afferent firing frequency (imp/s) measured under basal conditions (IP = 0 mmHg) before, and 1, 2, and 3 h after the first distension with PBS/BSA or BoNT/A. The results are presented as mean firing rate (imp/s). No significant difference at each time point (one-way analysis of variance (ANOVA) with post hoc Dunnett’s multiple comparison) after vs. before treatment. (**B**,**C**) Quantification of the afferent firing frequency measured in response to distension (IP = 0 to 50 mmHg before (green), and 1 (pink), 2 (red), and 3 (brown) h after the first distension with PBS/BSA (circles, (**B**)) or BoNT/A (triangles, (**C**)). The results are presented as delta mean firing rate (imp/s) as a function of IP, and correspond to the average of delta mean firing frequency (* *p* < 0.05, *** *p* < 0.001; 2-way ANOVA with post hoc Dunnett’s multiple comparison; after vs. before treatment). (**D,E**) Estimation of jejunal compliance before (green), and 1 (pink), 2 (red), and 3 (brown) h after the first distension with PBS/BSA (circles, (**D**)) or BoNT/A (triangles, (**E**)). The compliance was gauged by the pressure–volume relationship. The results are presented as jejunal volume (microliters, µL) as a function of IP, and correspond to the average of jejunal volume estimated. No significant difference at all time points (2-way ANOVA with post hoc Dunnett’s multiple comparison) after vs. before treatment. Number (N) of mice is indicated by 1 jejunum segment per mouse. Data are presented as mean ± SEM from 5 or 6 independent experiments.

In order to evaluate the effect of BoNT/A treatment on jejunal afferent nerve activity, each preparation was treated with a perfusate equivalent to 1 mL of a 3 nM BoNT/A solution or vehicle (PBS/BSA), adjusted to luminal volume. BoNT/A or vehicle was applied by intraluminal perfusion during three repeated distensions separated by a 15 min rest period without internal perfusion. Each jejunal segment was subjected to repeated distension (every 15 min) before, during, and after treatment with BoNT/A or PBS/BSA, and the afferent nerve activity was continuously recorded. Spontaneous afference nerve discharge (IP = 0 mmHg) measured from the nerves before treatment was similar for each group. This basal activity was maintained over time in the vehicle group, with a mean firing frequency of 28.80 ± 3.45 imp/s before treatment, and 36.24 ± 9.48; 32.1 ± 10.81, and 29.65 ± 11.26 imp/s 1, 2, and 3 h after vehicle application, respectively. The *p*-value was 0.89, 0.99, and 0.99 at 1, 2, and 3 h after vehicle application, respectively. However, this spontaneous activity tended to decrease over time after toxin application, with a firing frequency of 41.39 ± 12.58 imp/s before treatment, and 26.64 ± 12.64; 18.89 ± 9.93, and 10.45 ± 5.09 imp/s 1, 2, and 3 h after BoNT/A application, respectively (Figure 3A). The *p*-value was 0.66, 0.35, and 0.17 at 1, 2, and 3 h after BoNT/A application, respectively.

Regarding the mechanosensitive response, under vehicle conditions, jejunal distension evoked reproducible pressure-dependent increases in afferent nerve firing, with a weak but significant decrease from 2 h after treatment. For instance, at 50 mmHg, the mean firing frequency was 155.5 ± 18.3 imp/s before treatment, and 147.3 ± 17.1; 141.1 ± 20.5, and 123.3 ± 25.5 imp/s 1, 2, and 3 h after treatment, respectively (Figure 3B).

In contrast, after BoNT/A application, the level of multiunit discharge in response to distension showed a progressive and large decrease in jejunal afferent nerve firing frequency over time. The inhibition of this mechanosensitive afferent firing resulting from BoNT/A application was significant from 1 h after toxin perfusion, and was amplified after 2 and 3 h. For instance, in response to distension induced by an IP of 50 mmHg (maximum distension), the mean firing frequency was 128 ± 16.9 imp/s before BoNT/A treatment, and 105.3 ± 33.6; 48.9 ± 18.5, and 24.8 ± 11.2 imp/s 1, 2, and 3 h after treatment, respectively (Figure 3C).

To evaluate the evolution of muscle compliance over time, the jejunal volume was estimated at each pressure. There was no significant change in the pressure–volume relationship after PBS/BSA (Figure 3D) or BoNT/A (Figure 3E) application, indicating that there was no marked difference in the mechanical property of the gut wall after treatment.

**Figure 4:**
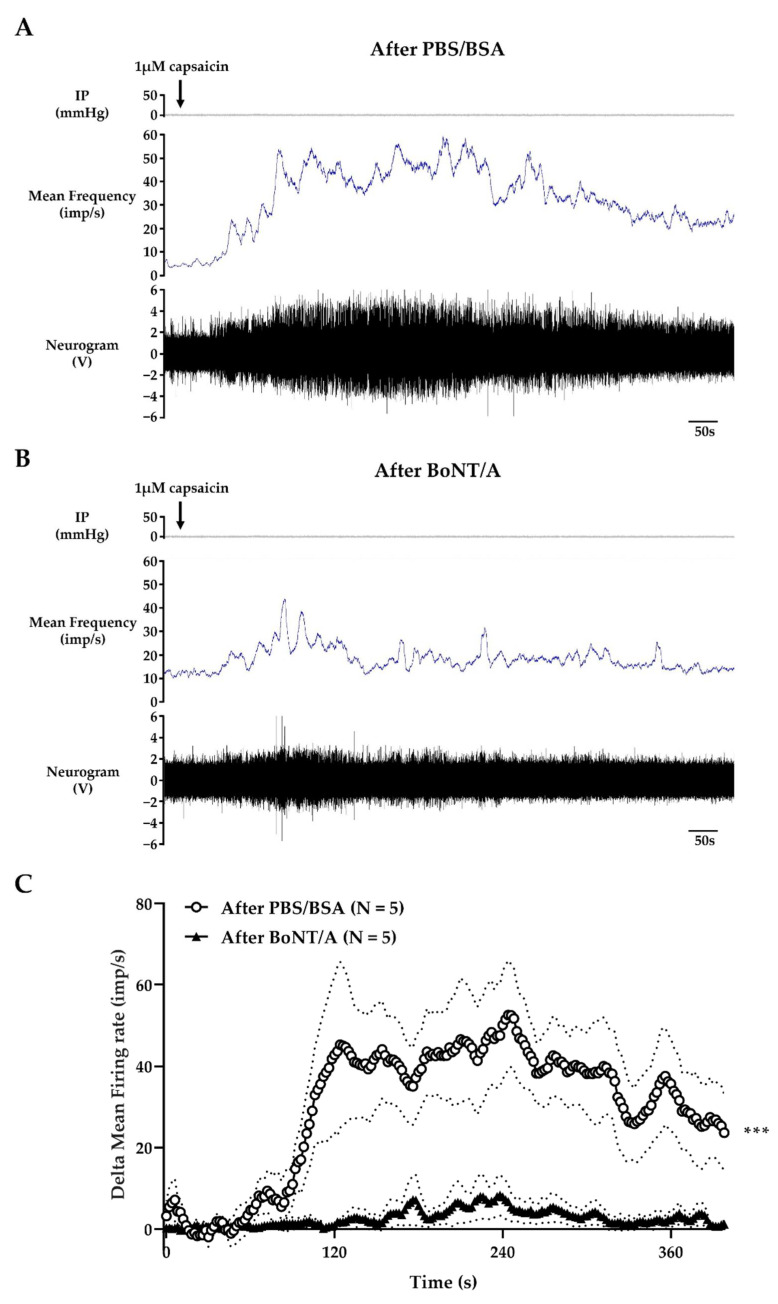
Effect of BoNT/A on multiunit discharge in response to 1 µM capsaicin. (**A**,**B**) Example of multiunit discharge recorded on mesenteric nerve in response to external perfusion of 1 µM capsaicin on (**A**) buffer- or (**B**) BoNT/A-treated jejunum segments. The internal pressure (IP; in mmHg) is presented in the top panel. The firing frequency (in imp/s) is presented in the middle panel, and in the bottom panel is the neurogram (in V). (**C**) Quantification of the afferent firing frequency measured in response to capsaicin perfusion 3 h after PBS/BSA or BoNT/A application. The results are presented as delta mean firing rate (imp/s) as a function of capsaicin perfusion time, and correspond to the average of delta mean firing frequency measured every 2 s. Number (N) of mice is indicated by 1 jejunum segment per mouse. Data are presented as mean ± SEM from 5 independent experiments for each treatment (*** *p* < 0.001; unpaired Student’s *t*-test; BoNT/A vs. PBS/BSA).

At the end of experiments, i.e., more than 3 h post-treatment, the afference nerve activity in response to TRPV1 stimulation was measured. In the vehicle-treated group, the external perfusion of 1 µM capsaicin at a rate of 80 mL/h generated a progressive increase in capsaicin concentration in the organ bath up to the final concentration of 1 µM, and generated a transient increase in multiunit discharge frequency, peaking 4 min after the start of capsaicin application (Figure 4A). Three hours after the first distension with BoNT/A, the response induced by capsaicin was considerably lower than in the post-vehicle treatment, with a maximum increase in firing frequency of 5.5 ± 3.5 and 44.5 ± 12 imp/s, respectively (Figure 4B,C).

In conclusion, BoNT/A inhibited the afferent nerve activity induced by distension, and also by stimulation of TRPV1, without a change in compliance.

### 2.4. BoNT/A Does Not Change the Quantity of Calcitonin Gene-Related Peptide in the Nerve Endings

**Figure 5:**
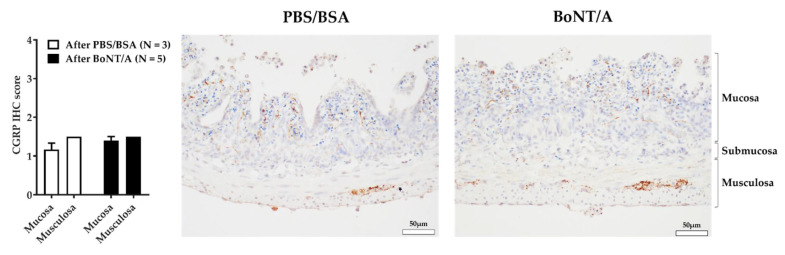
Immunostaining of calcitonin gene-related peptide (CGRP) in jejunum treated with PBS/BSA or BoNT/A. Staining levels (bar graph) and immunochemistry staining of CGRP in jejunum 3 h after PBS/BSA (**left**) or BoNT/A (**right**) treatment. Scale bar is 50 µm. Number (N) of mice is indicated by 1 jejunum segment per mouse. Data are presented as mean ± SEM from 3 or 5 jejunum preparations treated with PBS/BSA or BoNT/A, respectively.

In order to assess the impact of BoNT/A on the quantity of calcitonin gene-related peptide (CGRP) in the nerve endings, immunostaining was performed on sections from BoNT/A- and vehicle-treated preparations. CGRP staining was detected at the nerve endings in the mucosa and at the myenteric plexi. The level of CGRP was moderate, and similar in PBS/BSA- and BoNT/A-treated samples (Figure 5). The amount of CGRP in the nerve endings was therefore not affected by the application of BoNT/A.

## 3. Discussion

In this ex vivo study, we demonstrated, for the first time, that internal perfusion of BoNT/A during three successive jejunal distensions induced a time-dependent inhibition of multiunit discharge in response to mechanical and chemical stimulation.

A prerequisite of this study was to use a sensitive electrophysiological assay. The sensitivity of our system was validated with the use of a TRPV1 antagonist, CPZ, which induced a partial inhibition of multiunit discharge in response to distension. This result was in accordance with the effect of CPZ described in a previous electrophysiological study on the mouse jejunum [29]. Moreover, a strong inhibition of non- or mechanosensitive afferent activity could be measured following voltage-gated sodium channels (VGSCs) blockage by TTX. This is in agreement with the role of VGSCs as the main drivers of noxious signals from the viscera to the CNS, as supported by preclinical and clinical evidence [37,38,39].

The first objective of this study was to establish BoNT/A treatment conditions that allowed an internalization of the toxin. The cleavage of SNAP25 is an effective indicator of toxin activity, and therefore reflects the efficacy of the intoxication on jejunum/nerve preparations [40,41,42]. The intoxication after BoNT/A treatment (mucosal application of the toxin during three successive distensions) was evaluated with immunohistochemistry. The immunostaining of SNAP25 N-ter part (using an antibody that recognized its total and cleaved forms) showed the presence of a large and similar amount of this protein at the nerve endings of the mucosa and muscularis layers following both treatments. Regarding the cleaved form, as expected, c-SNAP25 was absent in the PBS/BSA condition, while staining was observed at the synapses after treatment with BoNT/A. It should be noted that this labelling did not allow discrimination between afferent and efferent nerve endings, but it did allow us to observe, under these treatment conditions, that BoNT/A had been internalized and had a significant SNAP25 cleavage activity.

The second objective was to analyze the effect of BoNT/A on the mesenteric nerve activity in response to mechanical and chemical stimulation. After PBS/BSA treatment, these experiments described a multiunit discharge on the mesenteric nerve under basal conditions (IP = 0 mmHg) and in response to distension, with a frequency comparable to that obtained before treatment. Only a slight decrease in firing frequency in response to distension was visible from 2 h after vehicle treatment, consistent with physiological fatigue. On the other hand, BoNT/A induced a progressive inhibition of the mesenteric afferent nerve activity. We observed that the spontaneous activity of the nerve tended to decrease over time. Moreover, the toxin application induced a significant time-dependent decrease in the frequency of multiunit discharge in response to mechanical stimulation. This effect on the mechanosensitive response appeared as early as 1 h after the first distension in presence of toxin. The effect was amplified 2 and 3 h after BoNT/A application; however, it is important to take into consideration that the effect at 2 and 3 h may be overestimated by the possible “physiological fatigue” described in our control condition. This time-dependent effect was in accordance with the mechanism of action of botulinum neurotoxin, and with previous results obtained from a bladder afferent nerve assay [27]. Interestingly, in jejunum segments previously incubated with BoNT/A, capsaicin-induced firing of the afferent fibers was drastically reduced, supporting an involvement of TRPV1-driven processes in BoNT/A’s quenching effects. Nevertheless, these data have to be considered cautiously, as these tissues were stimulated both mechanically and chemically in the same set of experiments, mainly for ethical reasons.

The mechanism of action (MOA) of BoNT/A on jejunal afferent nerve activity remains unclear. The toxin could have an effect at different levels of the nociceptive pathway [43]. An advantage of this ex vivo preparation is that we can limit the MOA to the peripheral compartment, without the involvement of the central nervous system, via retrograde transport [44]. However, this toxin could have an effect at one or more levels of the visceral nociceptive pathway, such as on stimulus detection or signal transmission.

As for stimulus detection, different elements must be considered. Regarding the mechanosensitive response, the toxin may interfere with the ability of mechanosensors to detect mechanical stimulation or/and modify the cellular localization of these proteins. One parameter that may alter the detection of mechanical stimulus was a change in jejunal compliance. Our results show that this parameter was not modified by BoNT/A, with no change in the pressure–jejunal volume relationship. Another hypothesis that can be put forward is that the toxin could influence the activation of mechanosensors, or their presence at the membrane. For example, this effect could be due to an impact on TRP channels. As a matter of fact, TRPV1 was characterized as being involved in jejunal/gut mechanosensation by the use of knock out (KO) murine models, or its antagonist, CPZ [29,31]. The use of KO mice demonstrated a peripheral mechanosensory role of TRPV1, with a reduction in colorectal mechanosensitivity [32]. Moreover, previous studies have shown that this toxin inhibits TRPV1 translocation to the membrane at the levels of trigeminal ganglion neurons and dorsal root ganglia [45,46,47,48,49]. An effect on the TRPV1 pathway could also explain the spontaneous nerve activity decrease following BoNT/A application. This decreasing tendency was previously described by Rong et al. in KO mice [29]. In addition, a change in TRPV1 cellular localization may explain the inhibitory effect of BoNT/A on the response induced by capsaicin. Furthermore, it has been reported that BoNT/A could interact directly with TRPV1 [50], and thus might structurally explain the blockade of capsaicin effect in our study. However, in view of the partial decrease in the mechanosensitive response in TRPV1 KO mice or induced by CPZ [29], the inhibition of this channel could only partially explain the effect of BoNT/A. Another TRP, TRPA1, could be involved in this effect of BoNT/A. This ion channel was also described as being involved in mechanosensation [51], and previous studies showed co-trafficking of TRPV1 and TRPA1 with an inhibitory effect of BoNT/A [45,49]. This hypothesis could be verified by an evaluation of the cellular localization of these channels, and how this correlates with c-SNAP25 in nerve endings.

An effect of BoNT/A on neurotransmitter release would also explain why the mechanosensitive and pharmacological responses were reduced. A previous study on isolated rat bladder showed an inhibition of the evoked release of CGRP from afferent nerve terminals following BoNT/A application [52]. This effect of BoNT/A was also described in a model of migraine pain, with an inhibition of CGRP release from activated sensory neurons [53]. The impact of BoNT/A on CGRP release was indirectly tested via the quantification of the neurotransmitter in the nerve endings. An inhibition of CGRP exocytosis following BoNT/A treatment could induce an accumulation of CGRP in nerve terminals [54]. This immunostaining did not show a difference of CGRP expression in nerve endings. However, a direct evaluation of CGRP release into the lumen with an enzyme-linked immunosorbent assay (ELISA) would have been a more effective functional test to confirm this result.

Using an electrophysiological approach, we described an assay adapted for the evaluation of the effect of BoNTs on visceral nociception. One of the most important advantages of the jejunum nerve assay is that it enables one to repeatedly measure a reproducible electrophysiological afferent response to jejunal distension for an extended period. It presents the possibility of measuring spontaneous multiunit discharge under basal conditions (IP = 0 mmHg), as well as in response to repeated mechanical stimulation both before and a couple of hours after BoNT/A treatment, in the same sample. In addition, the sensitivity of this assay allowed the characterization of a partial or a strong inhibitory effect on mechanosensitive multiunit afferent nerve discharge, as observed with CPZ and TTX. Sensitivity and sustained response over time were important criteria in the selection of our model. These criteria should allow us to discriminate between different BoNTs according to their kinetics and potency. One limit of this approach is the interval between each distension, which decreases the accuracy of assessing the kinetics of BoNTs. This time interval can be reduced down to 10 min [55], although this would make it difficult to discriminate between toxins with a low difference of potency. In conclusion, this assay is an effective tool to show the early effect of BoNT/A on afferent nerve activity.

Another limitation of this study would be its translationality to humans [56,57]. Even though no animal model can mimic human visceral pain perfectly, visceral pain models have allowed the study of the pathophysiology of the disease, as well as the efficacy of potential analgesics [58,59]. Moreover, while interspecies differences have been described in BoNT sensitivity [60], protein engineering can produce BoNTs with improved affinity for human receptors to bypass these discrepancies [61].

## 4. Conclusions

In conclusion, the mouse jejunal mesenteric nerve preparation was sensitive to BoNT/A at the afferent level in our study. Ex vivo models describing the effect of BoNT/A on visceral nerve activity usually focus on the lower urinary tract and, more precisely, the efferent innervation [62,63,64]; the afferent activity is much less explored ex vivo [27,65]. The ex vivo jejunum preparation used here allows one to explore the effect of botulinum neurotoxins on the afferent activity at the visceral/intestinal level in a quick and reproducible way, with a reduced number of animals required compared to in vivo experiments. It could be further used to evaluate the effect of other serotypes or recombinant modified neurotoxins, and to potentially detect promising candidates to help patients with visceral pain, as there is still room for improved treatments.

## 5. Materials and Methods

### 5.1. Drugs

BoNT/A used in this study was a recombinant botulinum neurotoxin type A1 produced at IPSEN Bioinnovation (Milton Park, United Kingdom). This neurotoxin has the same primary sequence and activity equivalent to natural BoNT/A1 [66]. Capsaicin (8-methy-*N*-vanillyl-6-nonenamide, TRPV1 agonist) and gelatin (Prionex^®^ highly purified type A) were acquired from Sigma-Aldrich, Chimie Sarl, St. Quentin Fallavier, France (M2028 and G0411). Capsazepine (*N*-[2-(4-Chlorophenyl)ethyl]-1,3,4,5-tetrahydro-7,8-dihydroxy-2H-2-benzazepine-2-carbothioamide; a TRPV1 receptor antagonist; CPZ) was purchased from Tocris Bioscience, Bristol, UK. Tetrodotoxin (tetrodotoxin citrate; Na+ channel blocker; TTX) was purchased from Abcam, Amsterdam, Netherlands (AB120055). Freshly diluted aliquots were used in all experiments. Stock solutions were made by dissolving drugs in distilled water for TTX, in DMSO for CPZ, and capsaicin was dissolved in a solution of 50% DMSO plus 50% ethanol. One aliquot of each compound was thawed at +4 °C before use and discarded after use; this excludes gelatin, which was stored at +4 °C and used up to 2 weeks.

### 5.2. Jejunum Segments Preparation

Nineteen non-fasted male C57BL6/N mice (5–6 weeks old; Janvier Labs) were anaesthetized with 3% isoflurane in 2% O_2_ (deep anaesthesia) followed by a check of no residual reflex after pinching of the toes, decapitation, and exsanguination. Four mice were used for CPZ experiments, 4 mice for TTX experiments, 6 mice for experiments with PBS/BSA, and 5 mice for experiments with BoNT/A. One jejunal segment per mouse was used. All experimental procedures were in accordance with animal ethics and well-being regulations, approved by the Ethics Committee of Ipsen Innovation (C2EA; registration number 32), and were conducted in compliance with the relevant animal health regulation in France (Council Directive No.2010/63/UE of 22 September 2010 on the protection of animals used for scientific purpose)

Segments of jejunum were dissected as described previously [28]. In brief, the abdomen was promptly opened, and the entire jejunum was excised and placed immediately in cold carbogenated Krebs–Henseleit (KH) solution (118 mM NaCl, 4.7 mM KCl, 1.2 mM KH_2_PO_4_, 25 mM NaHCO_3_, 1.2 mM MgSO_4_, 11 mM D-glucose, and 2.52 mM CaCl_2_). Segments of jejunum (approximately 3 cm in length) were sampled with a mesenteric neurovascular bundle at the center of the loop (Figure 1A). Preparations were transferred into a customized recording chamber, and continuously superfused at 80 mL/min with carbogenated KH solution, and kept at 35 °C. Following this, the sample was gently mounted between two tubes, with the duodenal end on the input tube and the ileal end on the output tube (Figure 1A). The input port was attached to an infusion pump, enabling the continuous perfusion of the lumen with carbogenated KH solution at a rate of 3 µL/s. The intraluminal pressure (IP) was continuously monitored with one pressure transducer (Opcobe, Living system) at each port.

### 5.3. Multiunit Discharge Recording on Mesenteric Nerve

As described previously [28], for each sample, the mesenteric nerve located between blood vessels was isolated by gently removing the fatty tissue, taking care not to damage the vessels and nerve. The tip of the suction electrode was positioned immediately next to the nerve, which was slowly aspirated into the capillary up to a sufficient length. In order to isolate the recording electrode and the nerve tip from the rest of the preparation, the glass capillary was closed with adipose tissue (mechanical sealing, Figure 1A). The glass suction electrode was connected to a Neurolog NL100AK headstage (IBIS Instrumentation, Ottawa, ON, Canada). Signals were amplified with an NL104 amplifier (gain 10K), a band pass filtered at 300–3000 Hz, and with an NL125 filter. The signal was automatically digitized and sampled at 20 kHz using a Micro1401 interface, and recorded and displayed on a PC running the Spike2 software package (Cambridge Electronic Design, CED, Cambridge, UK).

### 5.4. Mechanical Stimulation

To investigate the effect of drugs on jejunal mechanosensitivity, segments of jejunum were distended by a progressive increase in IP. This protocol was inspired by previous studies [29,55]. The increase in pressure was induced by the output port closing with a maintain ofinternal perfusion with Krebs solution at a rate of 3 μL/s to a maximal IP of 50 mmHg. After a resting period (45 min stabilization period), preparations were distended every 15 min. The first distensions were used to assess the basal response of the sample. If these 3–4 responses were not similar, the sample was discarded. The pressure–response relationship was determined using a custom-made script (provided by CED) in the Spike2 interface. Delta firing frequency corresponded to an afferent firing frequency measured at a given pressure minus the afferent firing frequency measured before distension in the absence of pressure. The jejunal volume at each IP was estimated. All data are expressed as mean ± SEM.

### 5.5. Pharmacological Stimulation

To evaluate the response to TRPV1 stimulation 3 h after intraluminal application of BoNT/A or vehicle, 1 µM capsaicin solution was applied on the same samples used to study the mechanosensitive response by bath superfusion at a rate of 80 mL/h, as described previously [29,67]. The concentration of capsaicin in the organ bath was progressively increased until it reached 1 µM. The nerve activity was measured continuously. Delta firing frequency corresponded to an afferent firing frequency measured over time after the start of capsaicin perfusion minus the baseline afferent activity over a 400 s period beforeperfusion. All data are expressed as mean ± SEM.

### 5.6. Treatments

In dedicated experiments 1 µM tetrodotoxin (TTX) or 30 µM capsazepine (CPZ) were applied by 5-min incubation in an organ bath before jejunal distension, and maintained during distension with external perfusion terminated. These distensions were preceded (15 min before) by a distension in the presence of the respective vehicle, ultrapure water (for TTX) or DMSO (for CPZ) applied in the same manner.

Previously, 3 nM BoNT/A was described as having an effect on bladder strip parasympathetic-dependent contractility [64]. Each jejunal segment was thus treated with a perfusate equivalent to 1 mL of a 3 nM solution, adjusted to luminal volume. BoNT/A was applied by intraluminal perfusion during 3 repeated distensions separated by a 15 min rest period without internal perfusion. The third distension was followed by a washout, with a restart of the intraluminal perfusion of KH, which was maintained throughout the rest of the experiment. Of note, 20 min before BoNT/A or vehicle treatment, samples were internally perfused with KH containing 0.5% gelatin to reduce non-specific binding of the toxin on tubing.

### 5.7. Immunohistochemistry

At the end of the ex vivo recording, jejunum samples (3 treated with PBS/BSA and 5 treated with BoNT/A) were immediately fixed in 10% *v*/*v* formalin (VWR Chemicals, France) at room temperature for 24 h. Jejunums were then cut into multiple cross-sections of 3 to 4 mm, dehydrated, embedded in paraffin blocks, and histological slides (5 µm) were finally prepared. For immunohistochemical staining of the tissue sections, a standard avidin–biotin–peroxidase procedure was used, as described in previous work [66]. After a heat-induced epitope retrieval step, endogenous peroxidases were blocked for 10 min in 3% H_2_O_2_. Slides were incubated with primary antibodies specific to the BoNT/A-cleaved form of SNAP25 (c-SNAP25, EF14007, rabbit polyclonal, IPSEN, France; overnight incubation), the N-ter part of SNAP25—thus recognizing both SNAP25 full and cleaved forms (111 011, Synaptic Systems, Göttingen, Germany; overnight incubation), or CGRP (C8198, Sigma-Aldrich, St Louis, MO, USA; incubation for 1 h). Sections were then incubated with a biotinylated anti-rabbit or anti-mouse IgG, secondary antibody for 30 min (Vector Laboratories, Burlingame, CA, USA), followed by a 30-min incubation with an amplification system (avidin–biotin) coupled to horseradish peroxidase (Vector Laboratories, Burlingame, CA, USA). Finally, sections were incubated for 5 min with 0.02% diaminobenzidine (DAKO, Carpinteria, CA, USA), and counterstained with hematoxylin.

To assess the expression of these proteins in the tissues, the level of their specific IHC staining was evaluated under a light microscope by a trained investigator (VM from the investigative pathology department), using a 5-point scale scoring system (0: no staining, 1: low staining, 2: moderate staining, 3: strong staining, 4: very strong staining).

### 5.8. Statistical Analysis

Statistical analysis was carried out using GraphPad Prism version 8.3.0 (GraphPad Software, San Diego, CA, USA). Data obtained with or without CPZ or TTX were compared using paired Student’s *t*-tests. Data for spontaneous nerve activity obtained before, and 1, 2, and 3 h after BoNT/A or vehicle application were compared using a one-way ANOVA and post hoc Dunnett’s multiple comparison test. Data for multiunit discharge frequency and jejunal volume obtained before, and 1, 2, and 3 h after BoNT/A or vehicle application were compared using a two-way ANOVA and post hoc Dunnett’s multiple comparison test. Data for capsaicin-induced responses obtained after BoNT/A application were compared to the PBS/BSA condition using unpaired Student’s *t*-tests. A *p*-value of less than 0.05 was considered as statistically significant.

### 5.9. List of Abbreviations

ANOVA, analysis of variance; BoNT/A, botulinum neurotoxin A; CGRP, calcitonin gene-related peptide; CPZ, capsazepine; DMSO, dimethyl sulfoxide; ELISA, enzyme-linked immunosorbent assay; imp/s, impulse per second; IP, intraluminal pressure; KO, knock out; N, number of mice; mmHg, millimeter of mercury; MOA, mechanism of action; PBS/BSA, phosphate-buffered saline/ bovine serum albumin; c-SNAP25, cleaved Synaptosomal-Associated Protein 25; t-SNARE, target soluble NSF (*N*-ethylmaleimide-sensitive factor; SEM, standard error of the mean; SV2, synaptic vesicle protein 2; TRPA1, transient receptor potential ankyrin 1; TRPV1, transient receptor potential vanilloid 1; TTX, tetrodotoxin; U.S. FDA, United States food and drug administration; V, volt

## Data Availability

The data presented in this study are available in this article.
